# Greater Survival Improvement in African American vs. Caucasian Women with Hormone Negative Breast Cancer

**DOI:** 10.7150/jca.39091

**Published:** 2020-02-21

**Authors:** Robert Wieder, Basit Shafiq, Nabil Adam

**Affiliations:** 1 Department of Medicine, Rutgers New Jersey Medical School, Rutgers Biomedical and Health Sciences; 2 The Cancer Institute of New Jersey, Rutgers Biomedical and Health Sciences; 3 Institute of Data Science, Learning, and Applications (I-DSLA), Rutgers University Newark; 4 Department of Computer Science, Lahore University of Management Sciences (LUMS); 5 Department of Management Science and Information Systems, Rutgers Business School

**Keywords:** breast cancer, African Americans, risk factor, survival

## Abstract

**Background:** African American women have not benefited equally from recently improved breast cancer survival. We investigated if this was true for all subsets.

**Methods:** We identified 395,170 patients with breast adenocarcinoma from the SEER database from 1990 to 2011 with designated race, age, stage, grade, ER and PR status, marital status and laterality, as control. We grouped patients into two time periods, 1990-2000 and 2001-2011, three age categories, under 40, 40-69 and ≥ 70 years and two stage categories, I-III and IV. We used the Kaplan-Meier and logrank tests to compare survival curves. We stratified data by patient- and tumor-associated variables to determine co-variation among confounding factors using the Pearson Chi-square test and Cox proportional hazards regression to determine hazard ratios (HR) to compare survival.

**Results:** Stage I-III patients of both races ≥ 70 years old, African American widowed patients and Caucasians with ER- and PR- tumors had worse improvements in survival in 2001-2011 than younger, married or hormone receptor positive patients, respectively. In contrast, African Americans with ER- (Cox HR 0.70 [95% CI 0.65-0.76]) and PR- (Cox HR 0.67 [95% CI 0.62-0.72]) had greater improvement in survival in 2001-2011 than Caucasians with ER- (Cox HR 0.81 [95% CI 0.78-0.84]) and PR- disease (Cox HR 0.75 [95% CI 0.73-0.78]). This was not associated with changes in distribution of tumor or patient attributes.

**Conclusions:** African American women with stage I-III ER- and PR- breast cancer had greater improvement in survival than Caucasians in 2001-2011. This is the first report of an improvement in racial disparities in survival from breast cancer in a subset of patients.

## Background

African American women have a lower incidence 1 but a persistently higher mortality from breast cancer than Caucasians 1-6. Spectrums of well documented patient-associated and tumor-associated variables have contributed to this disparity 7-21. African Americans are diagnosed at a younger age 19, higher stage 22, 23, higher grade 24, have a higher frequency of ER- and PR- tumors 22, 25, 26 and are more likely to be single than Caucasians 18, 22. All of these factors co-vary 21. In addition, well documented treatment-related disparities that include time to surgery, standard of care radiation, chemotherapy and social disadvantages 27 endow a worse prognosis in African American women than in Caucasian women with breast cancer.

Although overall mortality rates have declined in the last three decades, African American women did not benefit from this decline equally with Caucasians 1, 28-35. However, while population-averaged trends depict a uniform picture of unrelenting or progressively disparate outcomes, they systematically hide the impact of opposite trends, should they occur in population sub-segments 21. In one example, African American women with regional disease appeared to have superior increases in survival than Caucasians between the 1970s and 1990s, while no differences were evident in the unparsed population 1. In a study of the Surveillance, Epidemiology and End Results (SEER) data, the percentage increases in one-, three- and five-year survival of women diagnosed with all forms of breast cancer between the period 2004-2009 compared to the survival of patients diagnosed in the period 2001-2003 appeared to be consistently greater in African American women than in Caucasian women 36. Five-year age standardized survival for all stages combined also increased more between these two time periods for African American women than for Caucasian women 36. In another SEER study, when comparing the annual hazard rates for death in the two to seven years after initial diagnosis of women with locoregional ER- and to a lesser extent, ER+ breast cancer, decreases in the period 1997-2004 compared to those in the period 1990-1994 appeared to occur at a greater rate in African American women than in Caucasian women 37. Despite the apparent narrowing of differences in survival in patient subsets, disparities in the combined population persisted and continued to be reported. Although these studies did not address nor provide statistical treatment of the data, they nevertheless presented evidence that suggests that a greater improvement in survival may have occurred among some African American women with breast cancer than in Caucasian women.

Here, we undertake a formal investigation and focus on testing the hypothesis that African American women with locoregional ER- and PR- breast cancer had a greater improvement in survival after the turn of the century than their Caucasian counterparts. Stratification by ER and PR status is relevant, as it differentiates breast cancer categories of different cellular origin, biology, response to therapy, relapse pattern, prognosis and frequency in African American patients 21. We present the possibility that improvements in breast cancer survival have begun to chip away at racial disparities at least in one breast cancer category. We use the SEER database to stratify the population and compare patient survival Cox proportional hazard ratios (HR) between the two races while controlling for tumor and patient characteristics annotated in the SEER database.

## Methods

### Data and Patients

We analyzed the SEER database of the National Cancer Institute 38 from 1973 to 2011 (SEER database) for female patients with ICD-O-3 diagnostic codes for adenocarcinoma of the breast, as before 21, yielding 1,307,298 cases. The study was approved by the Rutgers Biomedical and Health Sciences Institutional Review Board-Newark.

We considered patients with race designated as White or Caucasian and Black or African American 21. We restricted the dataset analysis to newly diagnosed patients with stages I, II and III and patients who were classified as stage IV or recurrent, those with clear designations of tumor grades 1, 2 or 3, estrogen receptor status as positive (ER+) or negative (ER-), progesterone receptor status as positive (PR+) or negative (PR-), age, marital status as single, married, separated, divorced or widowed, and breast cancer laterality as right or left. For our analysis, we grouped ages into three categories, under 40 years, 40-69 years and ≥ 70 years, to roughly correspond to earlier age patients who get breast cancer less frequently, have more aggressive tumors and who do not have recommendations to receive population screening, to the population for whom screening is recommended by various organizations, and the older population for whom screening is not recommended and who tend to have less aggressive disease, respecttively. We analyzed 395,170 patients who were diagnosed between 1990 and 2011, because the database did not include ER and PR status before 1990.

### Statistical Analysis

We analyzed data from two time periods, 1990-2000 and 2001-2011. We defined survival in months as time from diagnosis of stage I, II or III breast cancer or from initial diagnosis of recurrence or presentation with metastatic disease to the time of death from any cause within the decade of analysis, since the cause of death in the SEER database is frequently listed as due to an immediate physiologic event and often not attributed to the underlying cause of breast cancer. Patients alive on December 31, 2000 and December 31, 2011 in the two analytic periods, respectively, were included in the analysis up to the respective periods' cut-off date and were censored from subsequent analysis.

We used the Kaplan-Meier method to generate survival curves and used the logrank test to compare the curves 39. We used Cox proportional hazards regression analysis to determine hazard ratios to compare survival. The predictor variables were the time periods between 1990 and 2000 and between 2001 and 2011, with the earlier decade as the base group. We stratified the data by race, age (in the three categories described above), stage, grade, ER status, PR status, marital status, and laterality as a negative control, and determined co-variation among these confounding factors using the Pearson Chi-square test. A p-value less than or equal to 0.05 was considered statistically significant. We computed estimates of Cox hazard ratios (HR) and associated 95% confidence intervals for each stratification from the 2001-2011 period versus the 1990-2000 period using the Cox proportional hazards regression model. All statistical analyses were performed using R Version 3.1.1 and R Studio Version 0.98.1056 (The R Foundation, Vienna, Austria) statistical software.

A major assumption of the Cox proportional hazards regression model is that the effect of a given covariate does not change over time, i.e., the hazards ratio remains constant over time 40, 41. To test if the proportional hazards assumption holds, we performed Schoenfeld residuals analysis. Non-proportionality of hazards is evidenced by the presence of a linear relationship of the Schoenfeld residuals against time 40. This linear relationship is tested by performing linear regression and determining the statistical significance of the regression coefficients based on their p-values. A p-value > 0.05 indicates a random pattern of residuals with time, implying that the proportional hazards assumption holds.

## Results

We compared the patient populations with stage I-III cancer and stage IV cancer to confirm that our data were similar to what is known about the characteristics of the two categories. A total of 95.9% of patients had localized disease and 4.1% had recurrent or metastatic disease. Median follow up for the periods 1990-2000 and 2001-2011 was 35 months and 51 months for stage I-III patients and 13 months and 19 months for stage IV patients, respectively.

All patient-associated and tumor-associated variables assessed had significantly different distributions in the two patient groups (**Table 1**). The stage IV group had higher proportions of patients who were African American, under 40, 70 or older, single or widowed than the stage I-III group. Patients in the stage IV group had higher frequency of tumors that were grade 3, ER- or PR- than patients in the stage I-III group. Laterality, which was included as a negative control, was not different between the two groups.

**Figure 1:**
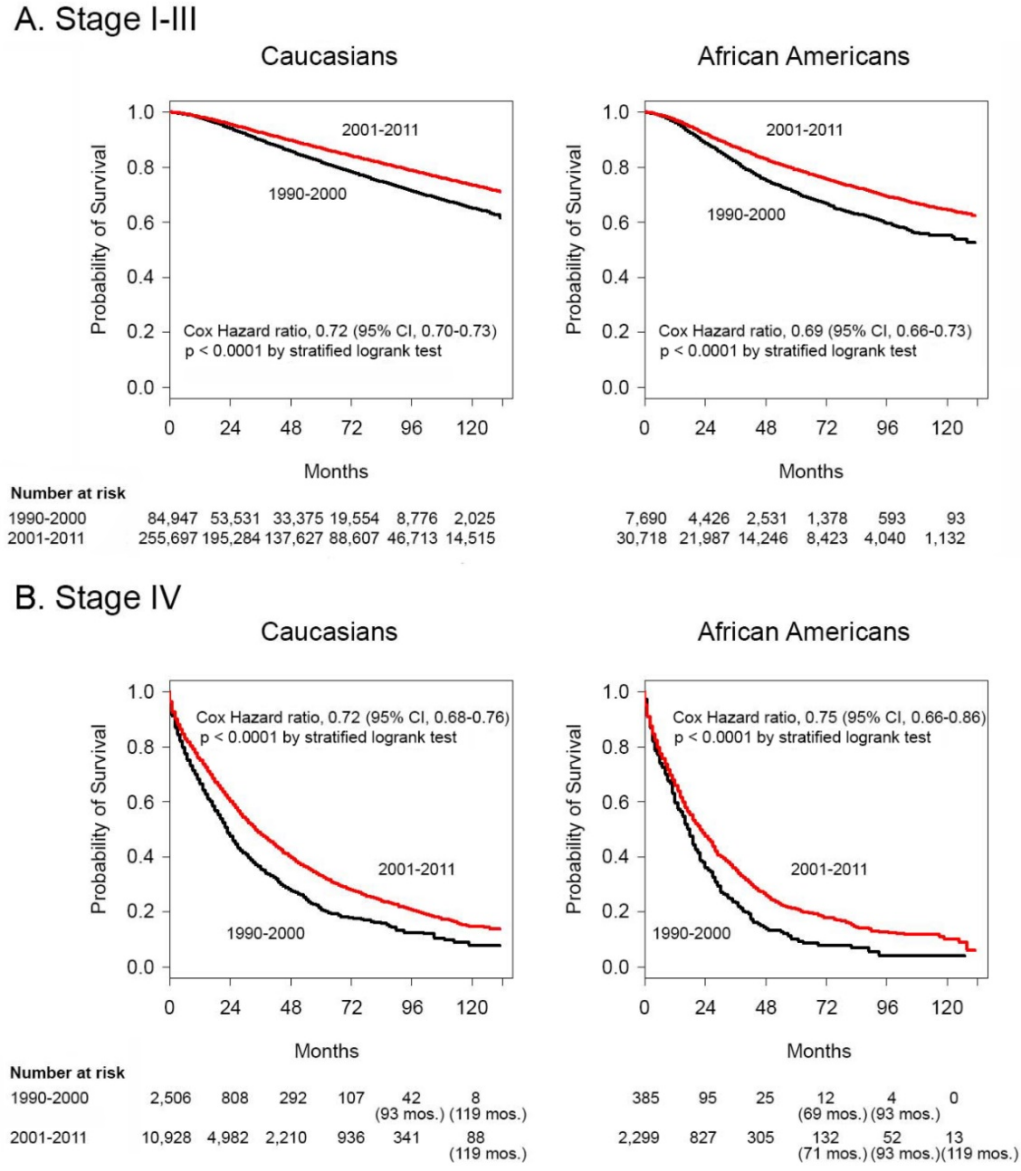
** Survival differences between 1990-2000 and 2001-2011 in African American and Caucasian women with stage I-III and stage IV breast cancer.** Kaplan-Meier survival curves for Caucasian patients and African American patients with **A.** stage I-III disease and **B.** stage IV disease diagnosed in the years 1990-2000 and 2001-2011. Differences in Cox hazard ratios were determined using the logrank test. Differences were considered significant at *P* ≤ 0.05. Patients at risk are shown at 0, 24, 48, 72, 96 and 120 months, except in some instances where there were no deaths and alternate times are inserted in parentheses.

We compared data from the two time periods straddling the turn of the century, 1990-2000 and 2001-2011. This provided a platform for analysis in the context of recent reports of improved survival from breast cancer 33-35. **Figures 1A** and **1B** depict Kaplan-Meier survival curves using logrank tests demonstrating significant improvements in Cox HR in the 2001-2011 time periods for Caucasians and for African Americans in both the stage I-III and stage IV groupings. Of note, the probability of survival of Caucasians continued to be better than that of African Americans. None of the groups with stage I-III disease reached median survival by 132 months after diagnosis in either decade. For stage IV patients, the median survival was 23 and 17 months in the 1990-2000 time period and 34 and 22 months in the 2001-2011 time period for Caucasians and African Americans, respectively.

To obtain added insight into differences in survival revealed by the Kaplan-Meier curves and the logrank test, we determined differences in distributions of patient- and tumor-associated characteristics between the two time periods for both stage I-III and stage IV patients using Chi square analysis. **Table 2** demonstrates significant differences in the distribution of both patient- and tumor-associated factors in the two time periods. Patients of African American race, patients between 40-69 and patients who were single were more frequently represented, while patients 70 and older and widows were less represented in the years 2001-2011 in both the stage I-III and stage IV categories. The frequency of grade 3 tumors also decreased in patients with localized and stage IV disease in the latter decade. Patients with stage III, ER- and PR- tumors decreased in the group with localized disease, but the frequency of ER- tumors did not change significantly and PR- tumors increased in stage IV patients in the years 2001-2011. There were no significant differences in the control variable of laterality. Given the significant changes in distribution of both patient- and tumor-associated characteristics in the two time periods, we reanalyzed survival differences using Cox proportional hazard ratios. While we found significant improvements in the Cox HR for survival in the latter decade in the unparsed populations of Caucasian and African American breast cancer patients and in all of the subpopulations stratified by various patient- and tumor-associated variables (**Table 3**), in most of these cases the proportional hazards assumption did not hold as reflected by the Schoenfeld residuals p-value < 0.05. Only in the case of Caucasians with Stage I (Cox HR 0.78 [95% confidence intervals 0.75 - 0.81]) and Stage IV disease (Cox HR 0.72 [95% confidence intervals 0.68 - 0.76]), African Americans with Stage I (Cox HR 0.71 [95% confidence intervals 0.63 - 0.81]), Stage II (Cox HR 0.73 [95% confidence intervals 0.67 - 0.79]) and Stage III disease (Cox HR 0.70 [95% confidence intervals 0.64 - 0.76]), patients in the < 40 years category (Cox HR 0.70 [95% confidence intervals 0.65 - 0.76]), patients with grade 1 tumors (Cox HR 0.80 [95% confidence intervals 0.75 - 0.85]) and patients in the Married (Cox HR 0.71 [95% confidence intervals 0.69 - 0.73]) and Divorced categories (Cox HR 0.84 [95% confidence intervals 0.79 - 0.89]) were the improvements in survival between the decades significant. There were also some notable differences between the extent of improvement in survival, specifically between Caucasian women with Stage II disease and Caucasians in the all stage category, women aged >70 and those in the other two age categories, Widowed women compared to Married or Divorced women and women with ER- and PR- tumors and patients with ER+ and PR+ tumors, respectively, but the linearity of the Schoenfeld residuals showed that the proportional hazards assumption did not hold. To remove the problem of non-proportionality 40, we further stratified the data.

We stratified the data by stage, race and the six patient- and the tumor-associated variables. The results of the Cox proportional hazards regression analysis with 3-variable stratification and 4-variable stratification are shown in **Tables 4 - 6**. **Table 4** demonstrates that Caucasian patients and African American patients had significantly lower Cox HR in the 2001-2011 time periods than in the 1990-2000 time periods in most patient- and tumor-associated variable categories. Several exceptions, including African Americans with Grade 1 disease, Caucasians and African Americans in the Separated category in the stage I-III groups and African Americans in the > 70 year, Grade 1, ER- and the Divorced and Widowed categories and Caucasians and African Americans in the Separated category in the stage IV grouping did not reach statistical significance due to low sample numbers (**Table S1**). The proportional hazards assumption did hold in all these cases except for Caucasians with Stage I-III disease who were < 40 years, had grade 3 disease and who were widowed, and for African Americans with Stage IV disease who had grade 3 disease and left sided laterality (**Table 4**).

Improvements in survival in the latter decade were significantly different in some subgroups than in others in the stage I-III disease category, where significant differences were noted with confirmed validity of the Cox HR determinations. Elderly Caucasians had much lower improvements in survival (Cox HR 0.88 [95% confidence intervals 0.85-0.91]) than Caucasians in the 40-69 year category (Cox HR 0.64 [95% confidence intervals 0.62-0.66]) and elderly African Americans had worse improvements in survival (Cox HR 0.83 [95% confidence intervals 0.75-0.93]) than African Americans in the < 40 year (Cox HR 0.58 [95% confidence intervals 0.49-0.68]) or the 40-69 year (Cox HR 0.67 [95% confidence intervals 0.62-0.72]) categories (**Table 4**, rows with grey shaded backgrounds). Similarly, African American widowed patients had worse improvements in survival (Cox HR 0.86 [95% confidence intervals 0.77-0.96]) than African American women who were single (Cox HR 0.68 [95% confidence intervals 0.60-0.76]) or married (Cox HR 0.61 [95% confidence intervals 0.55-0.67]) (**Table 4**, rows with grey shaded backgrounds). Caucasian patients with ER- (Cox HR 0.81 [95% confidence intervals 0.78-0.84]) and PR- tumors (Cox HR 0.75 [95% confidence intervals 0.73-0.78]) had much less improvement in survival in the latter decade than did Caucasians with ER+ (Cox HR 0.69 [95% confidence intervals 0.68-0.71]) and PR+ tumors (Cox HR 0.69 [95% confidence intervals 0.67-0.71]), respectively (**Table 4**, rows with grey shaded backgrounds). However, we did not find this difference in African American women with ER- and PR- tumors. In contrast, we found that there was a markedly greater improvement in the rates of survival in the latter decade in African American women with ER- (Cox HR 0.70 [95% confidences intervals 0.65-0.76]) and PR- disease (Cox HR 0.67 [95% confidences intervals 0.62-0.72]) than in their Caucasian counterparts (ER-, Cox HR 0.81 [95% confidence intervals 0.78-0.84], PR-, Cox HR 0.75 [95% confidence intervals 0.73-0.78]), with the proportional hazards assumption holding in both cases (**Table 4**, rows with orange shaded backgrounds). This was particularly significant in the setting of less improvement in the survival of Caucasians with ER- and PR- disease than of patients with corresponding hormone positive categories described above. None of the data in the stage IV group identified significant differences or trends in the improvement of the Cox HR between the two decades in any of the subgroups.

**Figure 2:**
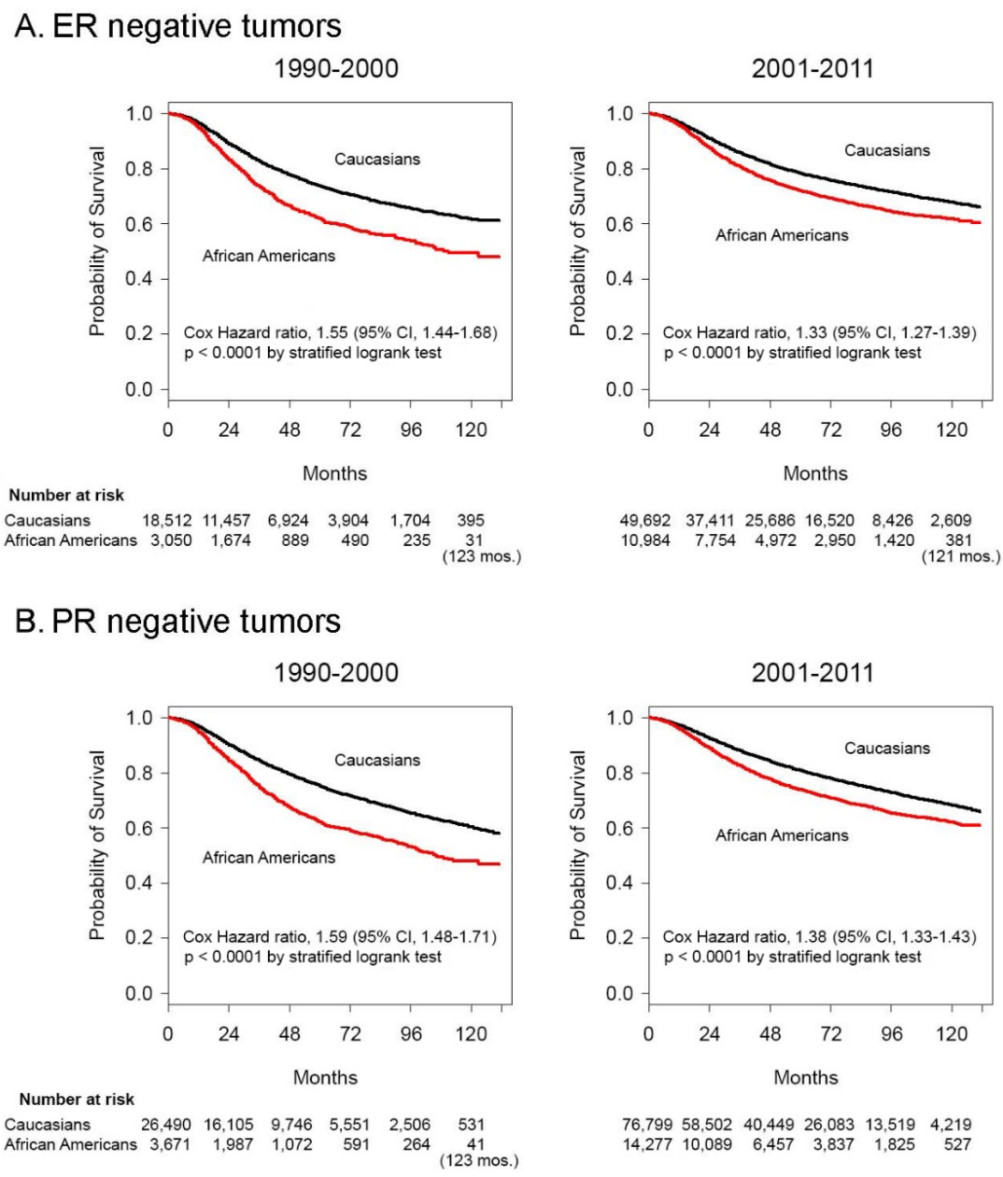
** Survival differences and between African American and Caucasian women with stage I-III hormone negative cancer in 1990-2000 and 2001-2011.** Kaplan-Meier survival curves for patients with Stage I-III disease with **A.** ER negative and **B.** PR negative tumors diagnosed in the years 1990-2000 and 2001-2011. Differences in Cox hazard ratios were determined using the logrank test. Differences were considered significant at *P* ≤ 0.05. Patients at risk are shown at 0, 24, 48, 72, 96 and 120 months, except in some instances where there were no deaths and alternate times are inserted in parentheses.

Kaplan-Meier survival curves and logrank test analysis demonstrated shrinking Cox hazard ratios comparing African Americans to Caucasians with stages I-III disease in the latter decade, for both ER- disease (Cox HR 1.55 [95% confidence intervals 1.44-1.68] in the 1990-2000 period vs. Cox HR 1.33 [95% confidence intervals 1.27-1.39] in the 2001-2011 period, *P* < .001) (**Figure 2A**), and PR- disease, (Cox HR 1.59 [95% confidence intervals 1.48-1.71] in the 1990-2000 period vs. Cox HR 1.38 [95% confidence intervals 1.33-1.43] in the 2001-2011 period, *P* < .001) (**Figure 2B**). The median survival of African Americans with stages I-III ER- tumors was 111 months (**Figure 2A**) and with PR- tumors was 106 months (**Figure 2B**) in the period 1990-2000 and was no longer reached for either group in the period 2001-2011. Caucasians with ER- and PR- tumors never reached the median survival in either decade.

These results indicate a significantly greater improvement in the Cox hazard ratios for survival of African American women with ER- and PR- breast cancer than that of Caucasians in the decade after the turn of the century. Nevertheless, survival of Caucasian women with ER- and PR- breast cancer remained greater than that of African Americans despite the documented superior improvement in survival in the latter group. We stratified the ER- and PR- data further to determine if potential changes in the distribution of tumor-associated and patient-associated co-variables could have contributed to improved survival favoring African Americans. For tumor-associated variables, we analyzed stage, grade and PR status in patients with ER- tumors and stage, grade and ER status in patients with PR- tumors. As in the unstratified data, some of the subcategories with sufficient elements exhibited significantly greater improvement in the Cox hazard ratio for survival in African American patients than in Caucasian patients in the decade after the century (**Tables 5** and **6**, rows with orange shaded backgrounds). African American women with ER- tumors had greater improvement in survival than Caucasian women with ER- tumors in the stage I category (Cox HR 0.66 [95% confidence interval 0.55-0.81] vs. Cox HR 0.89 [95% confidence interval 0.82-0.96]), in the PR- tumor category (Cox HR 0.67 [95% confidence interval 0.62-0.73] vs. Cox HR 0.79 [95% confidence interval 0.76-0.82] and in the Married category (Cox HR 0.62 [95% confidence interval 0.55-0.70] vs. Cox HR 0.77 [95% confidence interval 0.73-0.81], respectively (**Table 5**). African American women with PR- tumors had greater improvement in survival than their Caucasian counterparts in the > 70 year old category (Cox HR 0.74 [95% confidence interval 0.63-0.86] vs. Cox HR 0.90 [95% confidence interval 0.86-0.95], in the stage I category (Cox HR 0.61 [95% confidence interval 0.51-0.73] vs. Cox HR 0.80 [95% confidence interval 0.75-0.86], in the ER- category (Cox HR 0.67 [95% confidence interval 0.62-0.73] vs. Cox HR 0.79 [95% confidence interval 0.76-0.82], and in the Married category (Cox HR 0.59 [95% confidence interval 0.52-0.66] vs. Cox HR 0.71 [95% confidence interval 0.68-0.75], respectively (**Table 6**). African American women with PR- right sided tumors also has greater improvement in survival than their Caucasian counterparts in the latter decade (Cox HR 0.63 [95% confidence interval 0.57-0.70] vs. Cox HR 0.77 [95% confidence interval 0.73-0.80]. In all these noted cases, the proportional hazards assumption held.

Analysis of tumor-associated variables showed that there was a small decrease in the distribution of Caucasian patients with stage III disease in years 2001-2011 but no corresponding change in the distribution of African American patients with stage III disease in the ER- patient group (**Table 5**). Both Caucasians and African Americans had more grade 3 and PR- tumors that were ER- in the latter decade, suggesting a small increase in the aggressiveness of ER- tumors. In the PR- patient group, the stage differences were similar to the ER-group, indicating a small decrease stage III Caucasian patients in years 2001-2011 but without change in stage III African American patients (**Table 6**). There were slightly more Caucasian patients with grade 3 tumors but the increase in African Americans did not reach statistical significance (**Table 6**). African Americans with PR- tumors had a small decrease in the distribution of ER- tumors in the latter decade but the change in Caucasians was not significant. These data support the position that changes in these tumor-associated variables did not contribute to the significantly greater improvement in the Cox hazard ratios for survival of African American patients with ER- and PR- disease in 2001-2011 compared to the prior decade.

Analysis of patient-associated variables revealed that in both the ER- and the PR- stratified groups, there was a decrease in the under 40 year-old patient group but an increase in the single patient group in both Caucasians and African Americans (**Tables 5** and **6**). These data do not support contributions by changes in adverse patient prognostic characteristics to the disparate changes in survival. Combined with the lack of tumor-associated contributions, the data raise the possibility that perhaps, treatment-associated factors may be contributing to the differences in the changes in survival in patients with ER- and PR- tumors, countering conventional wisdom.

## Discussion

Our data demonstrate that, while most groups of patients with breast cancer had improved survival in the first decade of the century, African American women with ER- and PR- stage I-III disease had significantly greater improvement than their Caucasian counterparts. This stood in contrast to some subgroups who fared less well and had less improvement in survival than other patients in their respective stratification categories, including ER- and PR- Caucasian patients, elderly Caucasian and African American patients and widowed African American patients with stage I-III disease. These results support the fact that analyses by population averaging patients with a disease characterized by many variables will mask the existence of significant differences in certain subsets. In fact, our unstratified data showed overlapping confidence intervals for survival improvement spanning the two decades in African Americans and Caucasians for both groupings of stage I-III disease and stage IV disease. These results were in line with prior observations 5, 34. In one example, a study of the SEER Medicare databases that demonstrated no change in the absolute difference in survival between matched African Americans and Caucasians over 65 between 1991 and 2005 concluded that differences in survival appeared primarily related to presentation characteristics at diagnosis rather than treatment differences 5. Indeed, when we stratified the data by one variable or multiple variables, differences in survival became evident. Stratification is necessary in this type of analysis due to diverging distributions of variables with time and covariance among associated variables 21. The SEER database did not begin incorporating measurements for Her2/*neu*, another important prognostic variable, until 2010, so it could not be part of our analysis and presented a limitation on this investigation.

This study is the first to report greater improvement in survival in a subgroup of African American women with breast cancer than in their Caucasian counterparts. In contrast, other studies of different time periods, subgroup analyses and databases documented persistence of a stable 35, 42 or in some cases, a temporally progressive disparity in survival between African American and Caucasian women 28-31, 43. As we noted earlier, several studies that reported persistence of disparities in the overall populations presented data that, on closer inspection, suggested comparatively improved survival in subsets of African American women 1, 36, 37. While these studies did not note and did not analyze these observations, they provided the first hints for a greater increase in survival for a subset of African Americans.

With respect to the causes for the trends we observed, our analysis provides some potential insight. Further stratification of the ER- and PR- Caucasian and African American patients by the tumor-associated and patient-associated variables that we analyzed for our whole dataset did not support a role for these co-variables in the greater improvement in the survival of the hormone receptor negative African American population that we observed. On this basis, we hypothesize that perhaps treatment-associated variables may have played a role.

An interesting observation was a statistically significantly greater improvement in the hazard ratio for survival in the second decade for PR- African American compared with PR- Caucasian patients with right sided breast cancer **(Table 6)**. A similar result was not observed with patients with right sided ER- tumors **(Table 5).** This was likely due to a greater difference between the fraction of PR- African American and Caucasian patients whose tumors were also ER- **(Table 6)** than between the two groups with ER- tumors that were also PR- **(Table 5)**. This increase in the improved hazard ratio in the higher ER-/PR- population in **Table 6** paralleled the greater improvement in survival we documented with ER- and PR- tumors separately in African Americans. The fact that the difference was only observed in right sided tumors may be related to the differential effects of radiation therapy on right vs. left sided tumors on the long term adverse cardiovascular effects that result from the non-equal incidental radiation of the heart in left sided breast cancer 44-46. This likely negated the ER- and PR- tumor distribution effects on left sided tumors.

The causes of lower rates of improvement in the survival of elderly and widowed patients with localized disease and of Caucasians with ER- and PR- disease compared with those observed in younger patients and patients with ER+ and PR+ disease, respectively, may be multivariate. Standard guideline therapy, including surgery, are not administered to elderly patients at the same rate as to younger women 47-50. Co-morbidities in older women often result in faster deterioration following chemotherapy than in younger women and are significant factors in therapeutic decisions and survival 51. Perhaps the relative lack of progress in effective novel therapies for hormone receptor negative breast cancer left the ER- and PR- groups lagging behind the category of patients with hormone sensitive disease.

A possible reason for the greater improvement in the survival of African American patients with ER- and PR- disease is a general improvement in the rate of appropriate treatment of minority patients with localized breast cancer 52. A greater national focus on clinical trial participation by African Americans through programmatic efforts may have also raised the general awareness of appropriate treatment and cancer control 53, 54. Thus, we may not be witnessing an improved survival in appropriately treated patients but in fact, we may be seeing the effects of an improvement in the fraction of the African American population receiving appropriate treatment. The lower improvement in the Caucasian population with the same disease category may indicate that in fact, a limit of effectiveness of treatment for hormone receptor negative disease has been reached with current therapies and that African American patients are catching up to these limits. It is entirely possible that the bridging of disparities in survival in this subpopulation may reach a limit with current therapy due to reported biological differences responsible for more aggressive behavior of hormone negative cancer in African American women 55-59. Nevertheless, our study is the first to demonstrate that African Americans with ER- and PR- disease benefitted significantly more than Caucasians with ER- and PR- disease in the 21^st^ century. Clearly more effort is needed to eliminate social disparities 27, to understand disparities based on tumor biology and to target these molecular differences.

## Supplementary Material

Supplementary table S1.

## Figures and Tables

**Table 1:** Differences in the distribution of patient- and tumor-associated factors between stage I-III and stage IV breast cancer

Total Number stratified	Stage I-III		Stage IV		*P* (Chi square)
	379,052		16,118		
	Number	% in category	Number	% in category	
**Race**					
Caucasian	340,644	89.9	13,434	83.3	< .001
African American	38,408	10.1	2,684	16.7	
**Age grouping**					
< 40	23,257	6.1	1,116	6.9	< .001
40-69	257,169	67.9	10,320	64.0	
≥ 70	98,626	26.0	4,682	29.0	
**Grade**					
1	72,103	19.0	1,051	6.5	< .001
2	162,700	42,9	6,335	39.3	
3	144,249	38.1	8,732	54.2	
**ER Status**					
+	296,814	78.3	11,490	71.3	< .001
-	82,238	21.7	4,628	28.7	
**PR Status**					
+	257,815	68.0	9,289	57.6	< .001
-	121,237	32.0	6,829	42.4	
**Marital Status**					
Single	49,219	13.0	3.101	19.2	< .001
Married	224,023	59.1	7,587	47.1	
Separated	3,567	0.9	222	1.4	
Divorced	41,855	11.0	2,041	12.7	
Widowed	60,388	15.9	3,167	19.6	
**Laterality**					
Right	186,765	49.3	7,892	49.0	0.44
Left	192,287	50.7	8,226	51.0	

**Table 2:** Differences in the distribution of patient- and tumor-associated factors between the years 1990-2000 and 2001-2011 in patients with stage I-III and stage IV breast cancer

Number stratified	Stages I-III					Stage IV				
	1990-2000		2001-2011		*P* (Chi square)	1990-2000		2001-2011		*P* (Chi square)
	92,637		286,415			2,891		13,227		
	Number	% in category	Number	% in category		Number	% in category	Number	% in category	
**Race**										
Caucasian	84,947	91.7	255,697	89.3	< .001	2,506	86.7	10,928	82.6	< .001
African American	7,690	8.3	30,718	10.7		385	13.3	2,299	17.4	
**Age grouping**										
< 40	6,323	6.8	16,934	5.9	< .001	186	6.4	930	7.0	< .001
40-69	59,780	64.5	197,389	68.9		1,732	59.9	8,588	64.9	
> 70	26,534	28.6	72,092	25.2		973	33.7	3,709	28.0	
**Stage**										
I	45,598	49.2	143,627	50.2	< .001					
II	33,208	35.9	105,487	36.8						
III	13,831	14.9	37,301	13.0						
**Grade**										
1	14,526	15.7	57,577	20.1	< .001	150	5.2	901	6.8	< .001
2	41,011	44.3	121,689	42.5		1,032	35.7	5,303	40.1	
3	37,100	40.0	107,149	37.4		1,709	59.1	7,023	53.1	
**ER Status**										
+	71,075	76.7	225,739	78.8	< .001	2,063	71.4	9,427	71.3	0.92
-	21,562	23.3	60,676	21.2		828	28.6	3,800	28.7	
**PR Status**										
+	62,476	67.4	195,339	68.2	< .001	1,725	59.7	7,564	57.2	0.01
-	30,161	32.6	91,076	31.8		1,166	40.3	5,663	42.8	
**Marital Status**										
Single	10,407	11.2	38,812	13.6	< .001	441	15.3	2,660	20.1	< .001
Married	54,819	59.2	169,204	59.1		1,376	47.6	6,211	47.0	
Separated	591	0.6	2,976	1.0		20	0.7	202	1.5	
Divorced	9,575	10.3	32,280	11.3		318	11.0	1,723	13.0	
Widowed	17,245	18.6	43,143	15.1		736	25.4	2,431	18.4	
**Laterality**										
Right	45,394	49.0	141,371	49.4	0.06	1,439	49.8	6,453	48.8	0.33
Left	47,243	51.0	145,044	50.6		1,452	50.2	6,774	51.2	

**Table 3:** Cox Proportional hazards regression model for overall death in the years 2001-2011 compared with 1990-2000 in women with adenocarcinoma of the breast stratified by single variables of race, age grouping, stage, ER status, PR status, marital status and laterality and by race and stage.

Variables	Cox Hazard Ratio (Confidence Intervals)	*P* (Pearson Chi Square)	*P* (Scaled Schoenfeld residuals test)	Number of patients (1990-2000/ 2001-2011)
**Caucasians**				
All stages	0.76 (0.74 - 0.77)	< .001	< .001	87,453/266,625
Stage I	0.78 (0.75 - 0.81)	< .001	0.525	42,854/131,815
Stage II	0.70 (0.67 - 0.72)	< .001	0.023	29,922/ 92,324
Stage III	0.74 (0.71 - 0.77)	< .001	0.030	12,171/ 31,558
Stage IV	0.72 (0.68 - 0.76)	< .001	0.501	2,506/ 10,928
**African Americans**				
All stages	0.76 (0.72 - 0.80)	< .001	0.028	8,075/ 33,017
Stage I	0.71 (0.63 - 0.81)	< .001	0.852	2,744/11,812
Stage II	0.73 (0.67 - 0.79)	< .001	0.892	3,286/13,163
Stage III	0.70 (0.64 - 0.76)	< .001	0.478	1,660/ 5,743
Stage IV	0.75 (0.66 - 0.86)	< .001	0.046	385/ 2,299
**Age grouping - all patients**				
< 40	0.70 (0.65 - 0.76)	< .001	0.566	6,509/ 17,864
40-69	0.72 (0.70 - 0.74)	< .001	< .001	61,512/205,977
≥ 70	0.91 (0.88 - 0.93)	< .001	< .001	27,507/ 75,801
**Grade - all patients**				
1	0.80 (0.75 - 0.85)	< .001	0.502	14,676/ 58,478
2	0.81 (0.78 - 0.83)	< .001	< .001	42,043/126,992
3	0.79 (0.77 - 0.81)	< .001	< .001	38,809/114,172
**ER status - all patients**				
+	0.74 (0.73 - 0.76)	< .001	0.002	73,138/235,166
-	0.86 (0.83 - 0.89)	< .001	< .001	22,390/ 64,476
**PR status - all patients**				
+	0.74 (0.72 - 0.76)	< .001	0.015	64,201/202,903
-	0.81 (0.78 - 0.83)	< .001	< .001	31,327/ 96,739
**Marital status - all patients**				
Single	0.81 (0.76 - 0.85)	< .001	0.013	10,848/ 41,472
Married	0.71 (0.69 - 0.73)	< .001	0.166	56,195/175,415
Separated	0.85 (0.69 - 1.04)	0.120	0.024	611/ 3,178
Divorced	0.84 (0.79 - 0.89)	< .001	0.058	9,893/ 34,003
Widowed	0.92 (0.89 - 0.95)	< .001	0.002	17,981/ 45,574
**Laterality - all patients**				
Right	0.77 (0.75 - 0.79)	< .001	< .001	46,833/147,824
Left	0.77 (0.75 - 0.79)	< .001	< .001	48,695/151,818

**Table 4:**
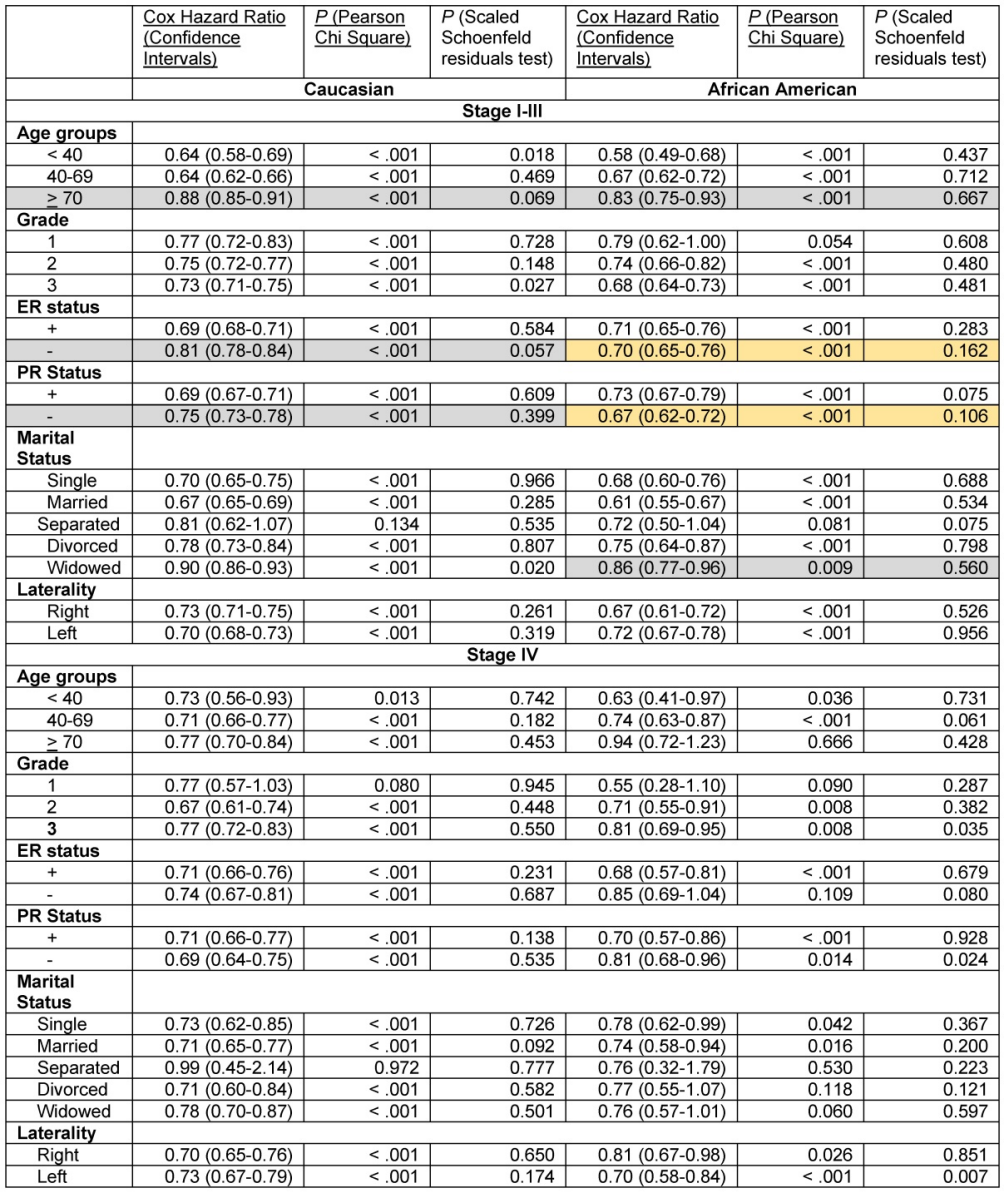
Cox Proportional hazards regression model for overall death in the years 2001-2011 compared with 1990-2000 in women with adenocarcinoma of the breast stratified by three variables, stage grouping, race, and by age grouping, grade, ER status, PR status, marital status and laterality.

**Table 5:**
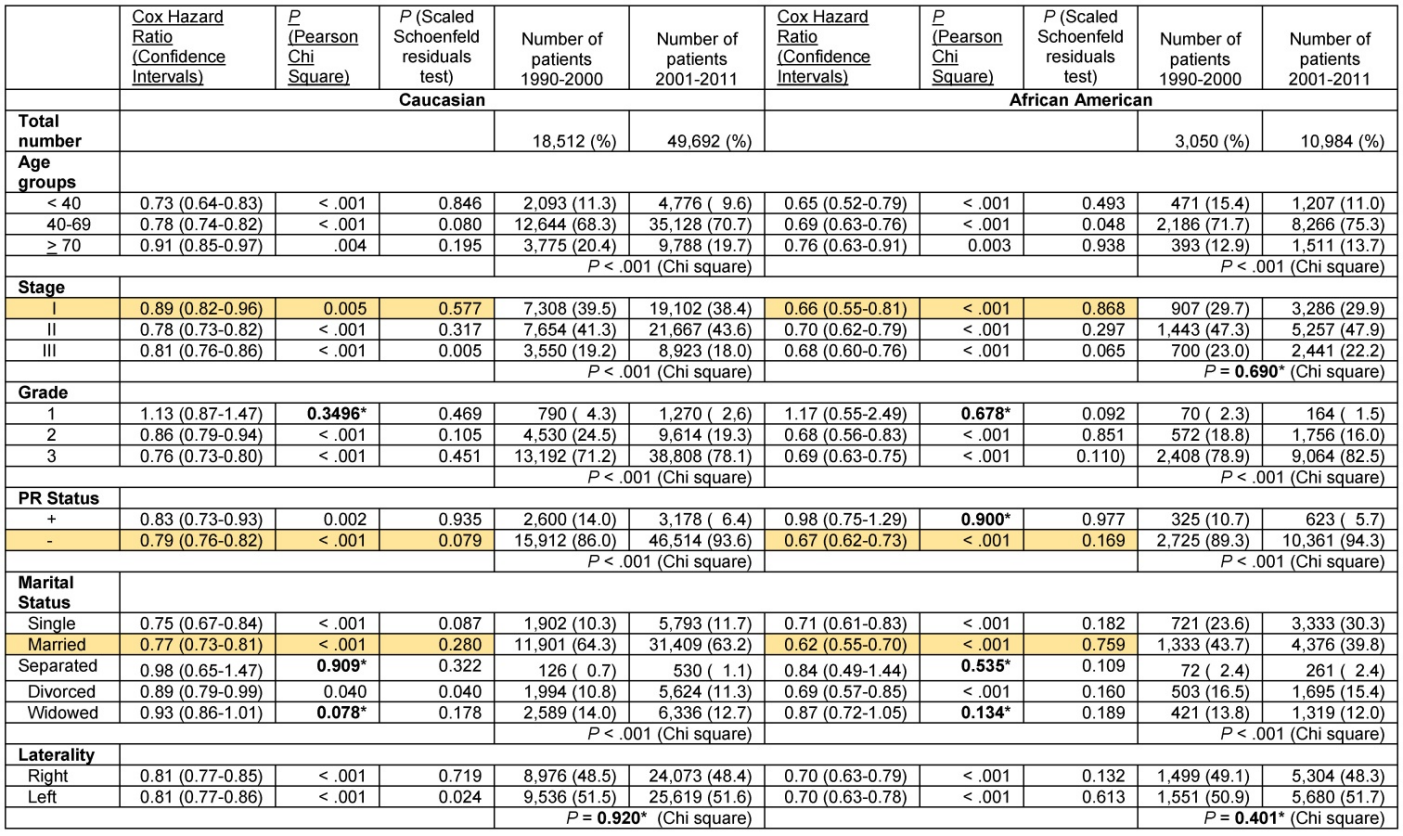
Cox Proportional hazards regression model for overall death in the years 2001-2011 compared with 1990-2000 in women with ER- adenocarcinoma of the breast stage I-III stratified by race, and by age grouping, stages I, II or III, grade, PR status, marital status and laterality.

*Not significant

**Table 6:**
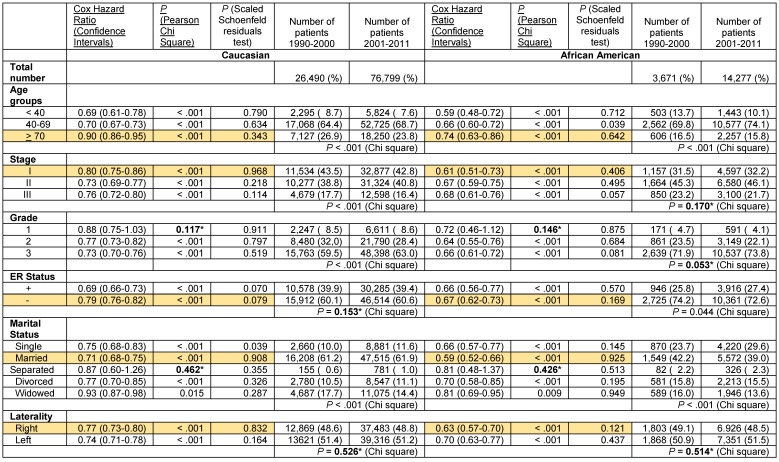
Cox Proportional hazards regression model for overall death in the years 2001-2011 compared with 1990-2000 in women with PR- adenocarcinoma of the breast stage I-III stratified by race, and by age grouping, stages I, II or III, grade, ER status, marital status and laterality.

*Not significant
