# Supplementary material for: Greater Survival Improvement in African American vs. Caucasian Women with Hormone Negative Breast Cancer
Source: J Cancer. 2020 Feb 21;11(10):2808–20. doi: 10.7150/jca.39091 (PMC7086262; doi:10.7150/jca.39091)
Supplement: Supplementary file 1 — Supplementary table S1. [file jcav11p2808s1.pdf]

Table S1. Number of patients in the years 1990-2000 and 2001-2011 with adenocarcinoma of the breast stratified by **stage**, **race**, **age grouping**, **grade**, **ER status**, **PR status**, **marital status** and **laterality**

	**All Patients (%)**		**Caucasian (%)**		**African American (%)**	
	1990-2000	2001-2011	1990-2000	2001-2011	1990-2000	2001-2011
			**Stage I-III**			
**All Patients**	92,637	286,415	84,947 (91.7)	255,697 (89.3)	7,690 (8.3)	30,718 (10.7)
**Age groups**						
< 40	6,323 (6.8)	16,934 (5.9)	5,399 (6.4)	14,207 (5.6)	924 (12.0)	2,727 (8.9)
40-69	59,780 (64.5)	197,389 (68.9)	54,527 (64.2)	175,045 (68.5)	5,253 (68.3)	22,344 (72.7)
≥ 70	26,534 (28.6)	72,092 (25.2)	25,021 (29.4)	66,445 (26.0)	1,513 (19.7)	5,647 (18.4)
**Grade**						
1	14,526 (15.7)	57,577 (20.1)	13,811 (16.3)	54,025 (21.1)	715 (9.3)	3,552 (11.6)
2	41,011 (44.3)	121,689 (42.5)	38,314 (45.1)	110,920 (43.4)	2,697 (35.1)	10,769 (35.1)
**3**	37,100 (40.0)	107,149 (37.4)	32,822 (38.6)	90,752 (35.5)	4,278 (55.6)	16,397 (53,4)
**ER status**						
+	71,075 (76.7)	225,739 (78.8)	66,435 (78.2)	206,005 (80.6)	4,640 (60.3)	19,734 (64.2)
-	21,562 (23.3)	60,676 (21.2)	18,512 (21.8)	49,692 (19.4)	3,050 (39.7)	10,984 (35.8)
**PR Status**						
+	62,476 (67.4)	195,339 (68.2)	58,457 (68.8)	178,898 (70.0)	4,019 (52.3)	16,441 (53.5)
-	30,161 (32.6)	91,076 (31.8)	26,490 (31.2)	76,799 (30.0)	3,671 (47.7)	14,277 (46.5)
**Combined ER/PR status**						
ER+/PR+	59,551 (64.3)	191,538 (66.9)	55,857 (65.8)	175,720 (68.7)	3,694 (48.0)	15,818 (51.5)
ER+/PR-	11,524 (12.4)	34,201 (11.9)	10,578 (12.4)	30,285 (11.9)	946 (12.3)	3,916 (12.8)
ER-/PR+	2,925 (3.2)	3,801 (1.3)	2,600 (3.1)	3,178 (1.2)	325 (4.2)	623 (2.0)
ER-/PR-	18,637 (20.1)	56,875 (19.9)	15,912 (18.7)	46,514 (18.2)	2,725 (35.5)	10,361 (33.7)
**Marital Status**						
Single	10,407 (11.2)	38,812 (13.6)	8,710 (10.3)	29,910 (11.7)	1,697 (22.1)	8,902 (29.0)
Married	54,819 (59.2)	169,204 (59.1)	51,573 (60.7)	157,321 (61.5)	3,246 (42.2)	11,883 (38.7)
Separated	591 (0.6)	2,976 (1.0)	425 (0.5)	2,274 (0.9)	166 (2,2)	702 (2.3)
Divorced	9,575 (10.3)	32,280 (11.3)	8,399 (9.9)	27,614 (10.8)	1,176 (15.3)	4,666 (15.2)
Widowed	17,245 (18.6)	43,143 (15.1)	15,840 (18.6)	38,578 (15.1)	1,405 (18.3)	4,565 (14.9)
**Laterality**						
Right	45,394 (49.0)	141,371 (49.4)	41,660 (49.0)	126,300 (49.4)	3,734 (48.6)	15,071 (49.1)
Left	47,243 (51.0)	145,044 (50.6)	43,287 (51.0)	129,397 (50.6)	3,956 (51.4)	15,647 (50.9)
			**Stage IV**			
**All Patients**	2,891	13,227	2,506	10,928	385	2,299
**Age groups**						
< 40	186 (6.4)	930 (7.0)	146 (5.8)	675 (6.2)	40 (10.4)	255 (11.1)
40-69	1,732 (60.0)	8,588 (64.9)	1,485 (59.3)	6,982 (63.9)	247 (64.2)	1,606 (69.9)
≥ 70	973 (33.7)	3,709 (28.0)	875 (34.9)	3,271 (29.9)	98 (25.4)	438 (19.1)
**Grade**						
1	150 (5.2)	901 (6.8)	135 (5.8)	808 (7.4)	15 (3.9)	93 (4.0)
2	1,032 (35.7)	5,303 (40.1)	928 (37.0)	4,548 (41.6)	104 (27.0)	755 (32.8)
**3**	1,709 (59.1)	7,023 (53.1)	1,443 (57.6)	5,572 (51.0)	266 (69.1)	1,451 (63.1)
**ER status**						
+	2,063 (71.4)	9,427 (71.3)	1,834 (73.2)	8,068 (73.8)	229 (59.5)	1,359 (59.1)
-	828 (28.6)	3,800 (28.7)	672 (26.8)	2,860 (26.2)	156 (40.5)	940 (40.9)
**PR Status**						
+	1,725 (59.7)	7,564 (57.2)	1,549 (61.8)	6,503 (59.5)	176 (45.7)	1,061 (46.2)
-	1,166 (40.3)	5,663 (42.8)	957 (38.2)	4,425 (40.5)	209 (54.3)	1,238 (53.8)
**Marital Status**						
Single	441 (15.3)	2,660 (20.1)	323 (12.9)	1,822 (16.7)	118 (30.6)	838 (36.5)
Married	1,376 (47.6)	6,211 (47.0)	1,259 (50.2)	5,504 (50.4)	117 (30.4)	707 (30.8)
Separated	20 (0.7)	202 (1.5)	11 (0.4)	140 (1.3)	9 (2.3)	62 (2.7)
Divorced	318 (11.0)	1,723 (13.0)	260 (10.4)	1,404 (12.8)	58 (15.1)	319 (13.9)
Widowed	736 (25.4)	2,431 (18.4)	653 (26.1)	2,058 (18.8)	83 (21.6)	373 (16.2)
**Laterality**						
Right	1,439 (49.8)	6,453 (48.8)	1,239 (49.4)	5,326 (48.7)	200 (51.9)	1,127 (49.0)
Left	1,452 (50.2)	6,774 (51.2)	1,267 (50.6)	5,602 (51.3)	185 (48.1)	1,172 (51.0)
